# Olfactory neuroepithelium biomarker platform: a proof from Alzheimer’s disease

**DOI:** 10.64898/2026.10.18.759991

**Published:** 2026-10-20

**Authors:** Koko Ishizuka, Guanqun Shang, Saba Malik, Carolyn Doty, Shrey Dave, Nicholas Rowan, Yukiko Lema, Andrew Lane, Akira Sawa

## Abstract

Unlike other diseases where target organs for biopsy are accessible, surrogate platforms are warranted for biomarkers of neuropsychiatric disorders due to the inaccessibility of the brain. We demonstrate a non-invasive platform combining a nasal soft-brush swab with single-cell Western blotting for neuron-specific protein analysis. Applied to Alzheimer’s disease targeting phosphorylated tau, this demonstrated superior diagnostic capacity compared with blood-based approaches, indicating that the olfactory neuroepithelium is a sensitive biomarker platform.

## Full Text Availability

The license terms selected by the author(s) for this preprint version do not permit archiving in PMC. The full text is available from the preprint server.

